# Structural Comparison of Hydroxyapatite from Clam Shell Waste and Eggshell Waste Compared to Commercial Synthetic Hydroxyapatite

**DOI:** 10.5704/MOJ.2411.004

**Published:** 2024-11

**Authors:** M Idulhaq, A Mudigdo, P Utomo, B Wasita, ME Trapsilantya

**Affiliations:** 1 Department of Orthopaedics and Traumatology, Universitas Sebelas Maret, Surakarta, Indonesia; 2 Department of Orthopaedics and Traumatology, Dr. Soeharso Orthopedic Hospital, Jawa Tengah, Indonesia; 3 Department of Pathology Anatomy, Universitas Sebelas Maret, Surakarta, Indonesia

**Keywords:** hydroxyapatite, clam shell, eggshell, biomaterial, bone repair

## Abstract

**Introduction:**

This study compares the quality of hydroxyapatite in Anadara granosa waste and laying chicken eggshell waste to commercial synthetic hydroxyapatite.

**Material and Methods:**

This experimental research included 27 samples of hydroxyapatite derived from clam shell waste (CSW-HAP), hydroxyapatite derived from eggshell waste (ESW-HAP), and commercial synthetic hydroxyapatite, with nine samples of each. The calcination method was used to process clam shell waste and eggshell waste into hydroxyapatite, which was then compared with synthetic hydroxyapatite from Bongros® for calcium and phosphate content. Scanning electron microscopy was used to compare their morphological structures.

**Result:**

The mean calcium levels in the CW-HAP, EW-HAP, and control groups were 41.3±2.9%, 41.5±2.3%, and 39.6±5.0%, respectively. According to One Way ANOVA, there was no significant difference between the CW-HAP or EW-HAP groups and the control group (p=0.49). The mean phosphate levels in the CW-HAP, EW-HAP, and control groups were 8.1±1.2%, 8.1±1.3%, and 9.4±2.0%, respectively. The results were also not significant (p=0.146).

**Conclusion:**

Clam shell waste and eggshells can be an alternative source of hydroxyapatite substitution, as demonstrated by the structural and porous formation of hydroxyapatite obtained from these sources (CW-HAP and EW-HAP) when compared to commercial synthetic hydroxyapatite.

## Introduction

Non-union fractures of long bones are a common orthopaedic condition that often require surgical intervention. They can result in delayed recovery, decreased quality of life, and negative impacts on psychological and socioeconomic status. The incidence of non-union fractures ranges from 5% to 10% and increases with the severity of the fracture^1^. Recently, several therapy options for non-union fractures have been developed. These include bone morphogenic protein, autologous bone grafting, platelet-rich plasma, heterologous bone grafting, low-intensity pulsed ultrasound, and the use of bone matrix such as calcium phosphate and hydroxyapatite^2,3^.

Hydroxyapatite (HAp) is a synthetic biomaterial that plays a crucial role in bone repair. The demand for synthetic hydroxyapatite (sHA) in bone graft applications is increasing due to its ability to promote bone regeneration and improve bone healing^4^. However, commercial synthetic HAp has significant drawbacks. Synthetic HAp has lesser crystallinity than naturally generated HAP, which can impact its mechanical characteristics and biocompatibility. Higher crystallinity is often connected with superior mechanical qualities and increased biocompatibility^5^. Commercial HAP is also unreasonably expensive. As a result, a cheaper natural material is needed^6^.

Every year, about ten million tons of mollusk shells are produced, with oyster, clam, scallop, and mussel shells accounting for more than 70%. Not only that, but roughly 7.2 million tons of eggshell trash are created every year, given that the eggshell is approximately 11% of the weight of an egg^7^. However, wasted shells are mostly dumped at sea or taken to landfills^8^. Both clamshells and eggshells are rich in calcium carbonate (CaCO_3_), which is the main component of their construction^9,10^. Calcium carbonate (CaCO_3_) is essential for the formation of hydroxyapatite (HAp), the primary inorganic component of human bones and teeth. The presence of CaCO_3_ in clam shell is necessary for the conversion to HAP^11^.

Shellfish and eggshell waste can be utilised to produce hydroxyapatite. Both clam shell and eggshell trash are tremendous in Indonesia. The goal of this study is to evaluate the quality of hydroxyapatite discovered in clam shell waste and eggshell waste with commercial synthetic hydroxyapatite (Bongros^®^).

## Materials and Methods

This study was conducted at Gadjah Mada University's medical department from October to November 2021. This study is divided into three groups: clam shell waste-derived hydroxyapatite (CW-HAP), eggshell waste-derived hydroxyapatite (EW-HAP), and a control group (Bongros^®^). In this study, the Federer formula was employed to calculate the sample size, which resulted in nine samples for each group. We used clean, fresh clam shell waste from Mariculture Farm - Tuban and eggshell waste from Diessa Farm - Yogyakarta. To optimise uniformity, our investigation employed only one source for each item.

Clam shell waste (CSW) and eggshell waste (ESW) derived hydroxyapatite was made through following steps. CSW and ESW were washed and dried. Subsequently, they were turned into powder and the powder was sieved using sieve with 270 mesh in diameter to obtain powder with a particle size 53 μm. The calcination process began, the powder was heated in 900°C for 4 hours to convert CaCO_3_ into CaO. CaO powder was combined with 0.5 M diammonium hydrogen phosphate and heated at 400°C until CW-HAP and EW-HAP powders were produced. Each CW-HAP and EW-HAP powder were mixed with sugar (with ratio 1:1) and compacted mechanically using CARVER Hydraulic model 3912 on 10 Mpa. Furthermore, the sintering process was conducted as long as 2 hours in 1300°C. The scanning electrical microscope (SEM) is applied to analyse the structure and morphology CW-HAP and EW-HAP.

The quality of structure and morphology of CW-HAP and EW-HAP was evaluated using SEM XRD (JEOL type JSM-636OLA). Moreover, the Ca/P CW-HAP and EW-HAP ratio were compared with commercial HAP (Bongros^®^).

All quality testing data were first evaluated for statistical purposes. The mean calcium and phosphate levels in each group were compared using statistical product and service solutions (SPSS). The Shapiro-Wilk test was used to check if the data distribution was normal. The comparison analysis was analysed using the one-way ANOVA test. If data normality cannot be achieved, the Kruskal-Wallis approach is used^12^.

## Results

**Fig. 1:**
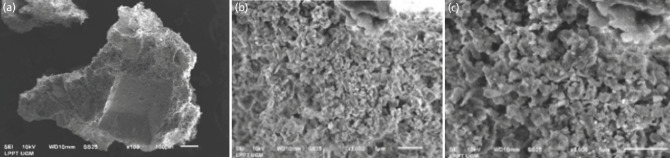
Spectrograph and the morphological structure of CW-HAP at 100, 3000, and 5000x magnification.

**Fig. 2:**
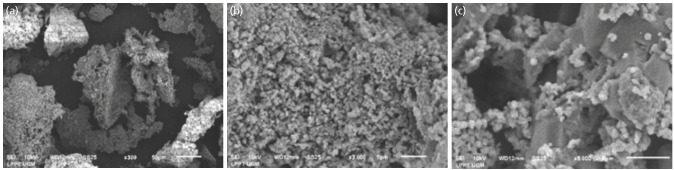
Spectrograph and the morphological structure of EW-HAP at 300, 3000, and 5000x magnification.

**Fig. 3:**
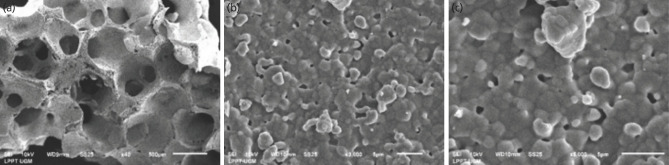
Spectrograph and the morphological structure of Bongros^®^ at 40, 3000, and 5000x magnification.

The microscopic imaging of CW-HAP was shown in Fig. 1, the imaging of EW-HAP was described in Fig. 2, while the imaging control group was shown in Fig. 3. From microscopic evaluation, it was determined that the particle size of CW-HAP (Fig. 1) and EW-HAP (Fig. 2) was smaller than the control group (Fig. 3).

The average calcium level in the CW-HAP and EW-HAP groups were 41.3±2.9% and 41.5±2.3%, respectively. The mean in the control group was 39.6±5.0%. According to One Way ANOVA, the difference between the CW-HAP and EW-HAP groups and the control group was not significant (p=0.49). The mean phosphate levels in the CW-HAP, EW-HAP, and control groups were 8.1±1.2%, 8.1±1.3% and 9.4±2.0%, respectively. The difference was not statistically significant (p=0.146). All results were shown at Table I.

EW-HAP and CW-HAP had smaller pore areas than Bongros^®^ (control group). Bongros^®^ had a lower pore volume than EW-HAP and CW-HAP. The control group had a lower pore surface area than EW-HAP and CW-HAP (Table I). Based on these findings, Bongros^®^ appeared to have a superior structure and morphology than EW-HAP and CW-HAP as bone graft constituents.

**Table I:** Comparison of calcium and phosphate level between EW-HAP, CW-HAP, and control group.

	CW-HAP	EW-HAP	Bongros^®^	Sig.
Calcium level	41.3± 2.9%	41.5± 2.3%	39.6± 5.0%	0.49
Phosphate level	8.1± 1.2%	8.1± 1.3%	9.4± 2.0%	0.146

**Table II:** Pore area, pore volume and pore surface area in EW-HAP, CW-HAP, and control group.

	CW-HAP	EW-HAP	Bongros^®^
Pore Area (nm)	5,222	5,436	6,265
Pore Volume (cm3/g)	0,0200	0,0167	0,0008
Pore surface area (m2/g)	2,813	2,147	0,3678

## Discussion

The ceramic structure of hydroxyapatite (Ca10(PO4)6 (OH)2) is safe for human use due to its similar mineral composition to human bone. Hydroxyapatite can be produced from both synthetic and natural sources, such as clam shells, eggshells, natural gypsum, and cow bones^13^. This study found no significant differences in calcium and phosphate content between CW-HAP, EW-HAP, and the control group.

The study's findings are consistent with those of Sakti and Magetsari, who discovered a link between the histological structure and radiograph diffractometer results of eggshell structure with synthetic HAp graft (Bongros^®^)^13^. Another study by Sari and Kurniawan demonstrated bone development when Anadara granosa was used as a replacement bone graft^14^.

Shellfish is a mineral composite and biopolymer composed of 95% to 99% CaCO_3_ crystals and minor quantities of oxides (0.696% SiO2, 0.649% MgO, 0.419% Al2O3, 0.33% SrO, 0.204% P2O5, 0.984% Na2O, 0.724% SO3) and 1-5% organic macromolecules. The aragonite layer on the skin's surface can be converted to the HAp layer using the hydrothermal technique in phosphate medium. HAp can be produced from shells and eggshells using solid reactions^15^. Hydroxyapatite was produced using a hydrothermal technique from CaO as a raw material in this study. To produce powdered hydroxyapatite, CaO powder was combined with 0.5M diammonium hydrogen phosphate and heated to 400°C. The hydroxyapatite was generated from clam shells (CW-HAP) and eggshells (EW-HAP).

Porous hydroxyapatite (HAp) is suitable for bone reconstruction^16^, as the formed pore serves as a medium for the growth of bone cell tissue^17^. In this study, locally produced CW-HAP and EW-HAP were classified as porous HAp. The data collected showed a structural correspondence between HAp and the formation of porous hydroxyapatite obtained from shells (CW-HAP) and from eggshells (EW-HAP) with synthetic hydroxyapatite, which is commonly used today (Bongros^®^). This is consistent with the literature review and meta-analysis, which indicate that eggshell HAp has high bioavailability and low toxicity^18^. This study supports the substitution of organic matter as a source of HAp and shows that CW-HAP and EW-HAP have promising mechanical properties, with similar properties compared to commercial hydroxyapatite, making them suitable for biomedical applications^19,20^. On the other hand, commercial HAp is typically synthetic and widely used in biomedical fields due to its bioactivity and biocompatibility. Commercial HAp may offer higher purity and controlled properties compared to CW-HAP or EW-HAP, but at a potentially higher cost. Both CW-HAP and EW-HAP, as a natural source, represent an environmentally friendly and sustainable alternative to commercial HAp, with potential for further exploration in medical applications.

This study provides a helpful comparison of hydroxyapatite obtained from clamshells and eggshells to commercially available synthetic hydroxyapatite. This strategy not only assesses the potential for these natural waste materials as alternatives, but it also supports sustainability by lowering reliance on resource-intensive synthetic manufacturing techniques. Eggshells and clam shells are easily available as waste materials, providing a scalable and accessible source of hydroxyapatite synthesis. This, together with the possibility for lower manufacturing costs, makes this a very practical and ecologically healthy strategy. Finally, the novel usage of these waste materials to generate a valuable resource with potential biomimetic features for healthcare applications distinguishes this study as an important addition to the field of sustainable material science.

We acknowledge a potential limitation in our study design related to selection bias. All data collection materials for this research originated from a single source. Relying on a single source for data collection materials may limit the generalisability of our findings. Future research could benefit from incorporating data collection materials from a wider range of waste sources to enhance the generalisability of the results.

## Conclusion

In conclusion, this study successfully synthesised hydroxyapatite (HA) from eggshell and clamshell waste (ESW/CSW-HA). The characterisation of ESW/CSW-HA revealed properties comparable to commercially available HA, suggesting its potential as a sustainable and cost-effective alternative for bone repair applications. Further in-vivo studies are necessary to evaluate the biocompatibility and efficacy of ESW/CSW-HA in promoting bone regeneration.
